# Profiling antibody epitopes induced by mRNA-1273 vaccination and boosters

**DOI:** 10.3389/fimmu.2024.1285278

**Published:** 2024-03-18

**Authors:** Bethany Girard, Elisabeth Baum-Jones, Rebecca L. Best, Thomas W. Campbell, Jack Coupart, Kyla Dangerfield, Abhilash Dhal, Michael Jhatro, Brian Martinez, Jack Reifert, John Shon, Minlu Zhang, Rebecca Waitz, Spyros Chalkias, Darin K. Edwards, Maha Maglinao, Robert Paris, Rolando Pajon

**Affiliations:** ^1^ Moderna, Inc., Cambridge, MA, United States; ^2^ Serimmune, Goleta, CA, United States

**Keywords:** antibody profile, COVID-19, dosing regimen, mRNA-1273, SARS-CoV-2

## Abstract

**Background:**

Characterizing the antibody epitope profiles of messenger RNA (mRNA)-based vaccines against SARS-CoV-2 can aid in elucidating the mechanisms underlying the antibody-mediated immune responses elicited by these vaccines.

**Methods:**

This study investigated the distinct antibody epitopes toward the SARS-CoV-2 spike (S) protein targeted after a two-dose primary series of mRNA-1273 followed by a booster dose of mRNA-1273 or a variant-updated vaccine among serum samples from clinical trial adult participants.

**Results:**

Multiple S-specific epitopes were targeted after primary vaccination; while signal decreased over time, a booster dose after >6 months largely revived waning antibody signals. Epitope identity also changed after booster vaccination in some subjects, with four new S-specific epitopes detected with stronger signals after boosting than with primary vaccination. Notably, the strength of antibody responses after booster vaccination differed by the exact vaccine formulation, with variant-updated mRNA-1273.211 and mRNA-1273.617.2 booster formulations inducing significantly stronger S-specific signals than a mRNA-1273 booster.

**Conclusion:**

Overall, these results identify key S-specific epitopes targeted by antibodies induced by mRNA-1273 primary and variant-updated booster vaccination.

## 1 Introduction

COVID-19 is caused by infection with SARS-CoV-2 and remains an ongoing public health threat (1). The development and widespread availability of COVID-19 vaccines, including previously authorized mRNA-1273 [SPIKEVAX; Moderna, Inc., Cambridge, MA, USA (2)], a messenger RNA (mRNA)-based vaccine encoding the prefusion stabilized spike (S) glycoprotein of the Wuhan-Hu-1 (ancestral) SARS-CoV-2 strain, has played a key role in the pandemic response (3–5). Despite the successes of early vaccines such as mRNA-1273 in reducing hospitalization and death caused by COVID-19 (3, 6, 7), antibody-mediated immune responses from primary vaccination wane over time (8, 9), and new variants have emerged with decreased susceptibility to vaccination against the ancestral strain (10, 11).

These initial challenges have been addressed in part by the addition of booster doses of the vaccine and the development of variant-updated booster vaccines (5, 12, 13). Notably, a third dose of mRNA-1273 induced higher neutralizing antibody titers than that observed after dose 2 of the primary series (14). Moreover, while antibody persistence also wanes following boosters over time, the decline is not as substantial as that seen following primary vaccination (10). However, the exact mechanisms underlying these differential immune responses after primary and booster vaccination are not well understood. In addition, several variant-updated mRNA-1273 vaccines have been developed to broaden protection; most recently, bivalent vaccines containing both ancestral and omicron strains were authorized for use as a booster dose in multiple countries worldwide (15–18). Vaccines containing variant strains were developed to induce immune responses against key neutralization sites of the mutated S protein within the variant SARS-CoV-2 strain. In a preliminary study, variant-updated vaccines administered as a booster dose approximately 6 months after the mRNA-1273 primary series increased neutralizing titers against the ancestral strain and several variant strains beyond the peak titers elicited after primary vaccination (9). Further, in a more recent randomized phase 3 trial, a booster dose of bivalent vaccine containing omicron BA.1 and ancestral SARS-CoV-2 strains elicited superior neutralizing antibody responses against omicron (BA.1) and non-inferior responses against the ancestral strain compared with the monovalent mRNA-1273 vaccine (19).

Characterizing the antibody response after mRNA-1273 and variant-updated mRNA-1273 vaccination is essential toward further understanding the immunologic robustness and breadth of these vaccines. This study therefore used Serum Epitope Repertoire Analysis (SERA) technology (20–22) on sera samples from clinical trial participants to determine the SARS-CoV-2 S protein epitope profiles induced by mRNA-1273 and variant-updated booster vaccination relative to the mRNA-1273 primary series, as well as the durability and effectiveness of immune responses elicited by these vaccines.

## 2 Materials and methods

### 2.1 Participant samples

Sera were collected from adult participants (aged ≥18 years) enrolled in an open-label interventional phase (Part B) of a phase 2 study (NCT04405076; mRNA-1273 booster vaccine participants) (14), where participants received two doses of mRNA-1273, followed by an mRNA-1273 booster dose approximately 6 to 8 months later or participants enrolled in different parts of a nonrandomized phase 2/3 clinical study (NCT04927065) (13, 23), where recipients of the mRNA-1273 vaccine in the coronavirus efficacy (COVE) trial were rolled over to receive a booster dose of a variant-updated vaccine approximately 6 months later (**Supplementary Table 1**). All participants provided informed consent and all study materials were approved by a central institutional review board (Advarra; Columbia, Maryland); the studies were conducted in accordance with the International Council for Technical Requirements for Registration of Pharmaceuticals for Human Use, Good Clinical Practice Guidance, and applicable government regulations. A total of 886 serum samples representing 180 SARS-CoV-2 vaccinated and boosted participants, each with longitudinal post-vaccination time points, were used in this analysis. Samples were retrieved from five cohorts based on the formulation of the vaccine booster: mRNA-1273 (ancestral, n = 40); mRNA-1273.351 (beta, n = 20); mRNA-1273.211 (ancestral and beta, n = 40); mRNA-1273.617.2 (delta, n = 40); and mRNA-1273.213 (beta and delta, n = 40). Blood samples used for analysis were collected at day 1 (baseline), day 57 (28 days after dose two of the primary series), booster day 1 (>6 months after dose 2), and booster day 29. Samples were collected at additional time points for some booster formulations (indicated in **Supplementary Table 1**).

### 2.2 SERA assay

Presence of antibody binding epitopes in serum samples was characterized using the standard SERA assay that has been described in detail previously (20). SERA can semi-quantitatively assess the immune epitope repertoire in individuals for a broad range of exposures, including SARS-CoV-2 infection and vaccination, and can also assess changes in individual repertoires over time. Thus, we used SERA to summarize cohort-level changes in response to primary series and booster vaccination.

Serum was incubated with a randomized *Escherichia coli* peptide library displaying unique peptides of 12 amino acids (1 × 10^10^, 10-fold oversampled) at a 1:25 dilution in a 96-well, deep well plate. Antibody-bound bacterial clones were selected using 50 μL Protein A/G Sera-Mag SpeedBeads (GE Life Sciences, #17152104010350) immunoglobulin (Ig)G or by incubation with biotinylated anti-human IgM antibody (Jackson ImmunoResearch, #709-066-073) at 1:100 dilution, followed by incubation with 50 μL Dynabead MyOne Streptavidin T1 conjugated magnetic beads (IgM) (Thermo-Fisher, #65602). Selected bacterial pools were resuspended in growth media and incubated overnight at 37°C, 300 rpm to propagate. Plasmid purification, polymerase chain reaction (PCR) amplification, and DNA barcoding with well-specific indices was performed as described previously (20). Sample concentrations were normalized to 4 nM for each pool and analyzed with Illumina NextSeq500. Healthy control standards were included for every 96-well plate of processed samples to evaluate reproducibility, and these were not used to compute specific peptide enrichment (21).

### 2.3 S protein RBD ELISA

All serum samples were processed by an S protein receptor binding domain (RBD) enzyme linked immunosorbent assay (ELISA). SARS-CoV-2 S RBD (alpha variant strain) immobilized on 96-well plates (Nunc MaxiSorp) at 0.5 µg/mL were incubated overnight at 4°C. Following incubation, plates were washed with PBS and blocked with 5% non-fat milk in PBS for 2 hours at room temperature. Plates were then incubated with serum (1:250 dilution) for 1 hour at room temperature before being washed and incubated with anti-human IgG-HRP conjugated secondary antibody (Jackson ImmunoResearch) at 1:10,000 in blocking reagent for 1 hour at room temperature. After washing, the reaction was developed with exposure to 3,3’,5,5’-tetramethylbenzidine (TMB) reagent (ThermoFisher) for 15 minutes and then stopped with 1M hydrochloric acid. Absorbance was measured on a Tecan SPECTRAFluor Plus plate reader at 450 nm (21).

### 2.4 IMUNE-based motif discovery

Identifying Motifs Using Next-Generation Sequencing Experiments (IMUNE) is an algorithm that was applied to discover motifs specific to samples in different booster vaccine formulations (22). IMUNE motifs were assessed for enrichment in individual samples without regard to protein sequence, and the motifs were subsequently aligned to protein sequences where possible. Booster day 29 samples for each of the different booster vaccine formulations were compared to matching subject samples before primary vaccination (day 1 or baseline) and at >6 months after primary vaccination (booster day 1) to discover S protein-specific motifs that were not previously identified or motifs that were specific to particular booster formulations. Enrichment comparisons were performed for all IMUNE discovered motifs to identify epitopes that are specific to boosted samples.

### 2.5 PIWAS analysis

Published Protein-based Immunome Wide Association Studies (PIWAS) methodology, which detects linear epitopes based on SERA data, was used to identify antigen and epitope signals against the SARS-CoV-2 proteome (Uniprot reference: UP000464024) (24). PIWAS analysis was performed on individual samples, with comparisons drawn longitudinally for each participant and among different booster vaccine formulations using a large convenience control cohort (n = 2766) for signal normalization. Approximately 1 to 3 million 12-mers per sample were obtained from the SERA assay and decomposed into 5-mers and 6-mers. The enrichment score for each k-amino acid (k-mer) was calculated by dividing the number of unique 12-mers containing the k-mer divided by the number of expected k-mers for the sample (based on amino acid proportions) as previously described (21). Each PIWAS score at a specific amino acid represents an average score within a 5 amino acid frame using the tiling data of 5-mers and 6-mers spanning the sequence. The z-score was calculated on comparisons of values from the sample with those from the large convenience cohort control (n = 2766). Antigens were ranked using the Mann–Whitney U false-discovery rate (FDR) and tiling data was generated for top-ranked antigens in both sample and control data. The 95th quantile bands were calculated for each population and the most prominent S protein epitopes were identified.

### 2.6 PIE analysis

Protein-wide Identification of Epitopes (PIE), which analyzes and aggregates individual PIWAS signals using outlier statistics, was used to identify statistically significant shared regions along the S protein at the cohort level (25). PIE methodology for epitope identification was performed by locating epitopes that had stronger signal in the study samples relative to control data from the large cohort population (n = 2766). The distribution of sample values relative to the control was analyzed for each position on the S protein. Outlier threshold was calculated according to Q_75 _+ 1.5∗(Q_75_−Q_25_), where Q_x_ is the x^th^ percentile of the control values at that specific sequence location. The outlier sum statistic was approximately the sum of signal above the outlier threshold in the study samples. A null distribution for the outlier sum value was calculated by scrambling case/control labels and recalculating many times. A *P* value for the study samples and control comparison was calculated based on the null distribution, with the significant value set to 0.001 and the outlier sum threshold was set to the 99.5th percentile value of all positions, with an FDR of *P*≥0.001. All sequence locations that exceeded both thresholds were included in the final plot. Further details on the outlier sum calculation and FDR are described elsewhere (26, 27). The relevant pseudo code for IMUNE, PIWAS, and PIE are available upon request.

### 2.7 Paired PIE analysis

Paired PIE was used to determine changes in outlier signals for individual participants across two time points and quantify the proportion of antibody signal toward shared regions identified by PIE. Paired PIE compares outlier signals derived across paired time point samples (eg, baseline and day 57) for individual participants. Outlier signals are those that have PIWAS tiling values (eg, differences between baseline and day 57 for paired PIE analysis) above an outlier threshold, which is defined as 1.5 times the interquartile range above the 75^th^ percentile value of a cohort (28). The distribution of differences in PIWAS signal was first calculated for each subject on a non-S protein control portion of the SARS-CoV-2 proteome and the outlier thresholds were defined individually for each subject to determine an increase or decrease to outlier signal:


$$T_{I\;}^{i}=I_{75}^{i}+1.5\cdot \left(I_{75}^{i}-I_{25}^{i}\right)$$

$$T_{D\;}^{i}=D_{75}^{i}+1.5\cdot \left(D_{75}^{i}-D_{25}^{i}\right)$$

Increased and absolute value decreases across time points were calculated for each participant and compared to the thresholds to determine if signals were classed as outlier increase or decrease.

### 2.8 Additional statistical approaches

A two-sided Mann-Whitney U test was performed for each booster vaccination group (mRNA-1273.351, mRNA-1273.211, mRNA-1273.617.2, mRNA-1273.213) compared to mRNA-1273. Effect size was measured by Cohen’s d (29). Fraction positive of an epitope was measured by the proportion of samples with values above an outlier threshold defined as 1.5 times the interquartile range above the 75th percentile value (28) and fraction positive of a motif was calculated as the proportion of samples with a motif z-scores ≥4.

## 3 Results

### 3.1 Participant samples

A total of 696 serum samples were collected from 174 clinical trial participants who received an mRNA-1273 primary vaccination series and a subsequent booster vaccination with mRNA-1273 (Wuhan-Hu-1 [ancestral strain] only), variant-updated mRNA-1273.351 (beta strain only), mRNA-1273.211 (ancestral strain and beta), mRNA-1273.617.2 (delta strain only), or mRNA-1273.213 (beta and delta) (**Supplementary Table 1**). Overall, sera from 174 participants were analyzed at pre-booster time points (day 1 [baseline, before first vaccination] and day 57 [28 days after dose 2]), booster day 1 [>6 months after dose 2 and immediately before the booster]), and a post-booster time point (booster day 29), including sera from participants who received a booster vaccine with either mRNA-1273 (n = 40), mRNA-1273.351 (n = 20), mRNA-1273.211 (n = 37), mRNA-1273.617.2 (n = 39), or mRNA-1273.213 (n = 38).

### 3.2 Primary mRNA-1273 vaccination elicits strong antibody responses toward multiple SARS-CoV-2 S-specific epitopes that wane over time

We first identified significantly elevated SARS-CoV-2 S antibody binding epitopes after mRNA-1273 primary vaccination using PIWAS and PIE (see Methods). Samples were individually tiled along the sequence of the S protein (Wuhan-Hu-1) to show individual and shared signals.

**Figure 1:**
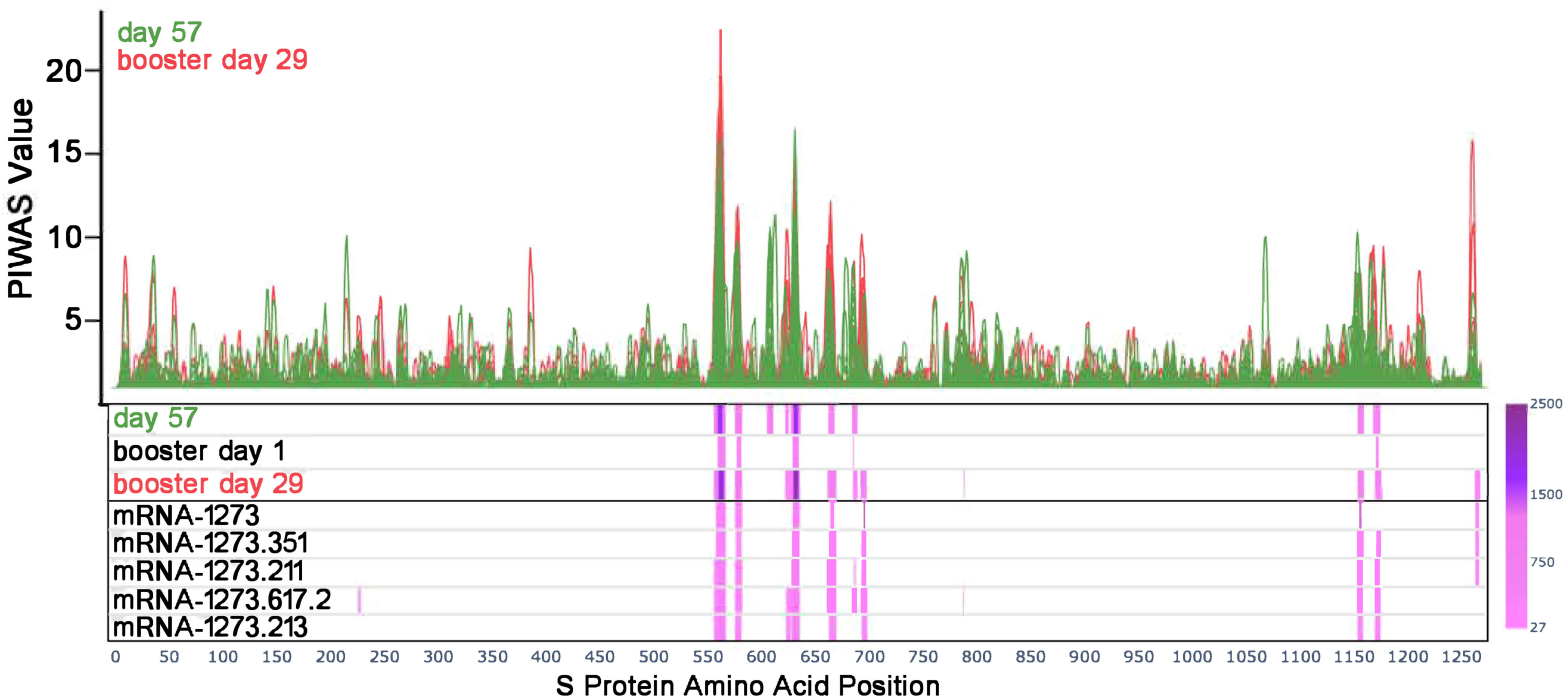
Antibody responses toward SARS-CoV-2 S-specific epitopes after primary and booster vaccination. PIWAS tiling plots at day 57 in green (28 days after dose 2 of primary mRNA-1273 vaccine series) and booster day 29 in red (28 days after booster vaccination) along the amino acid sequence of the SARS-CoV-2 S protein on the x-axis. Below the tiling plot are heat map visuals along the S protein sequence (x-axis only), depicting shared epitopes with statistically significant shared outlier sums as calculated by paired PIE (see Methods and Materials section 2.7). Significant regions are colored in a pink to purple gradient based on the magnitude of outlier sum as depicted in the color range bar on the right. The heat map rows show outlier sum significance of epitopes at day 57, booster day 1 (>6 months after dose 2), and booster day 29 versus baseline signal. Each booster vaccine formulation is also shown for comparison of significant epitopes detected. PIWAS, protein-based immunome wide association studies; S, spike.

Prior to vaccination (day 1), the majority of participants (169/174) showed low or no detectable signals toward SARS-CoV-2 epitopes. One participant had a strong signal toward many S-specific and nucleocapsid epitopes, while four other participants had a notable signal at baseline. At 28 days after dose 2 of the primary mRNA-1273 vaccination series (day 57), eight epitopes with significantly shared signals toward the S protein were identified in the full cohort of 174 participants (**Figure 1**), all of which aligned with entries in the immune epitope database (30). Significantly shared signals here refer to significantly shared outlier signals within a sample cohort (day 57) compared to a control cohort (day 1). **Table 1** lists the eight shared epitopes identified by PIWAS and PIE with the corresponding motifs identified by IMUNE (see Methods) aligned to these S protein regions, where the motifs are contained within the epitope sequences. At >6 months after dose 2 of the primary series (booster day 1), the signal toward S-specific epitopes was reduced compared with day 57, with fewer participant samples contributing toward this signal. Of the eight significant epitopes with shared outlier signals identified at day 57, five (558-KFLPFQQFGRD, 577-RDPQTLE, 624-IHADQLTPTWRVYS, 685-RSVAS, 1165-DLGDISGI) had reduced signal strength and prevalence at booster day 1 and the remaining three (607-QVAVLY, 662-CDIPIGAGI, 1151-ELDKYF) were no longer significantly shared. These results highlight that primary mRNA-1273 vaccination elicits robust antibody responses that progressively wane after the initial vaccination time points.

**Table 1:** Identified SARS-CoV-2 S-specific epitopes with significant outlier signals after vaccination.

Protein region (amino acid)	Epitope sequence	Effect size			Fraction positive			Aligned motifs	Fraction positive		
		day 57	Booster day 1	Booster day 29	day 57	Booster day 1	Booster day 29		Day 57	Booster day 1	Booster day 29
558-568^a^	KFLPFQQFGRD	1.6	0.5	1.16	0.72	0.22	0.57	KxLPFQQ, **[FML]xQFGR**	0.75	0.24	0.54
577-583^a^	RDPQTLE	1.32	0.39	0.95	0.57	0.11	0.41	RxPx[ST]L[DE]	0.54	0.03	0.30
607-612^b^	QVAVLY	0.66	0.13	0.02	0.28	0.05	0.06				
624-637^a^	IHADQLTPTWRVYS	1.52	0.6	1.45	0.7	0.21	0.67	TPxWRVY, **T[PA]Txx[IV]Y**	0.64	0.15	0.67
662-670^b^	CDIPIGAGI	0.52	0.13	0.72	0.24	0.09	0.34	**DIPIGAG**	0.10	0.01	0.26
685-689	RSVAS	0.45	0.24	0.22	0.2	0.09	0.13	RS[ILMV]AS	0.17	0.05	0.06
693-698	IAYTMS	0.33	–	0.58	0.09	–	0.22	YTxSLG, **IAYxMS**	0.08	–	0.22
788	^c^KQIYKTPP	0.25	–	0.22	0.07	–	0.08	KTPP[ILM]	0.02		0.01
1151-1156^a,b^	ELDKYF	1.02	0.28	0.98	0.39	0.1	0.36	FxxELxx[WY]F	0.81	0.2	0.73
1165-1172	DLGDISGI	0.72	0.26	0.67	0.25	0.1	0.28	DxSGI	0.42	0.07	0.37
1259-1263	DDSEP	0.15		0.4	0.08		0.16				

Non-shaded rows represent the initial eight significant epitopes identified at day 57 by PIE; gray shaded rows include epitopes with increased signal at booster day 29 versus day 57. Motifs in bold text were newly identified in boosted samples from this study.

S, spike.

a Previously reported neutralization epitopes (31, 32).

b Epitopes identified as significant outliers at day 57, but signal is with no longer significant at booster day 1.

c Added flanking sequence of peak signal location.

**Figure 2:**
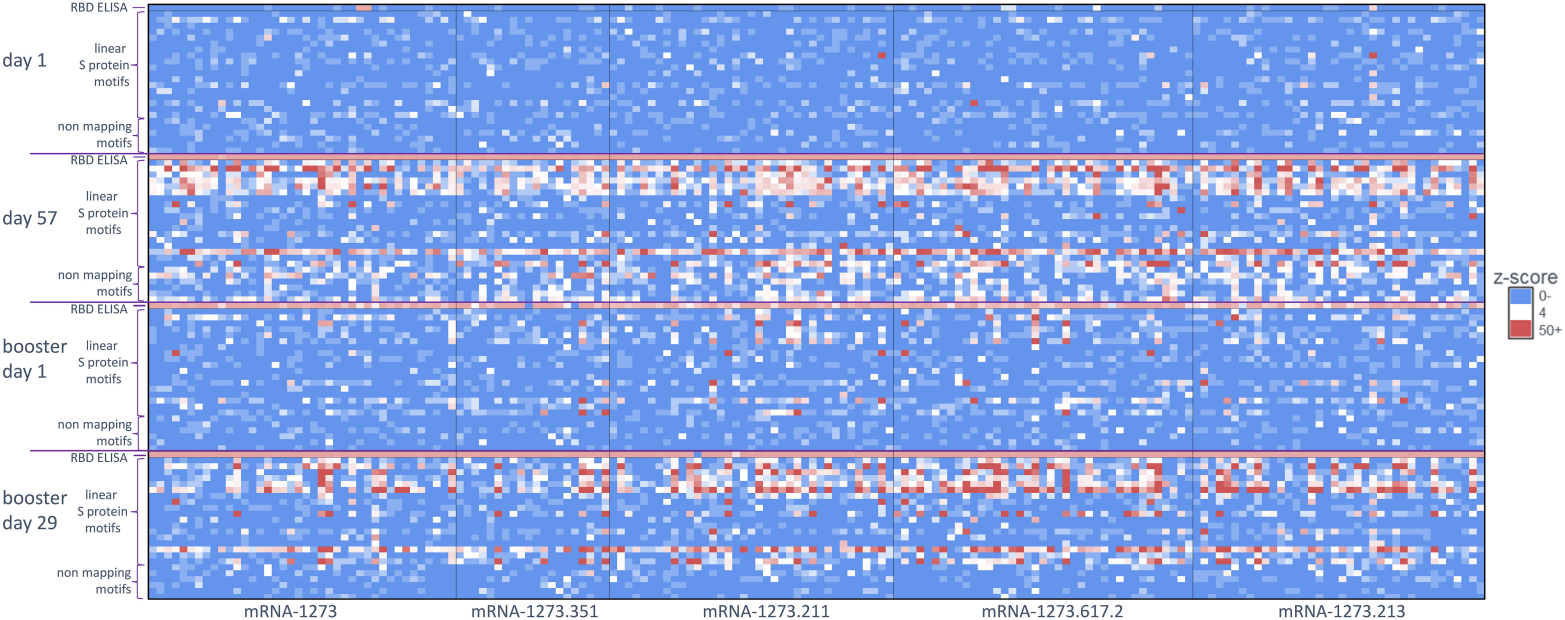
Antibody signal toward SARS-CoV-2 S-specific motifs after primary and booster vaccination. A heatmap of S protein motifs (rows) identified at baseline (day 1), 28 days after primary vaccination (day 57), >6 months after primary vaccination (booster day 1), and 28 days after booster vaccination (booster day 29). Z-scores for all 174 participants are shown (columns) and aligned to compare across vaccination time points. Different booster vaccine formulations are boxed. The top row and bottom six rows for each time point show the z-scores for the RBD ELISA and six non-mapping motifs respectively. RBD, receptor binding domain; S, spike.

For this study, we utilized a comprehensive and validated SARS-CoV-2 motif panel developed using IMUNE (21) to analyze antibody signals toward S-specific motifs, motifs aligned to other SARS-CoV-2 proteins, and non-mapping but SARS-CoV-2–specific motifs after primary vaccination. A heatmap showing z-scores for S-specific and non-mapping motifs for all samples across time points and by booster formulation is shown in **Figure 2**; longitudinal plots of z-scores for aligned motifs are shown in **Supplementary Figure 1**. These non-mapping motifs were discovered and validated as specific to SARS-CoV-2 infection or vaccination but the sequences of the motifs do not align exactly to SARS-CoV-2 (or any other Betacoronavirus) proteome. These motifs may represent structural mimics of antibodies targeting S proteins. The capture of such motifs that do not align exactly to the proteome of SARS-CoV-2 demonstrate the ability of the platform to capture additional antibodies beyond linear epitope–targeting antibodies alone. After primary vaccination (day 57), certain motifs or motif groups showed positive signal in only a small percentage of the full cohort of participant samples (n = 174); for example, a motif that aligned to the SARS-CoV-2 S protein at amino acid start sequence of 693 only accounted for 8% of participant samples. Motifs that aligned to SARS-CoV-2 S protein at amino acid start sequences of 577, 1148, and 1168 were more abundant, accounting for 54%, 81%, and 42%, respectively, of participant samples (**Table 1**). A motif group that aligned to amino acid start sequence 814 (KRSFIEDLLF) showed positive signal (z-score ≥4) in 21% of the day 57 samples, which was likely not well detected using PIWAS/PIE methods due to the multiple spans of “X” amino acids positioned in the motifs (eg, R[EHS]xxExxLF, KxSxIEx[ILM], and [ILMV]ExxLFxR); these “X” amino acids are likely not energetically important for antibody binding and represent gaps in the peptide sequences that reduce signal detection using tiling methods. A group of six non-mapping motifs collectively had a strong antibody signal in 64% of participant samples at day 57 (**Figure 2**; **Supplementary Figure 1**). Overall, the motif panel revealed a strong signal detected in primary vaccination samples (day 57) towards multiple S-directed motifs.

### 3.3 Booster vaccination restores the strong antibody response toward S-specific epitopes

A booster dose of mRNA-1273 or a variant-updated vaccine restored signal strength toward the S-specific epitopes identified by PIWAS/PIE analyses at day 57 (**Figure 1**), with these shared epitopes showing a similar signal at booster day 29 as seen on day 57. While most waning signals increased after booster vaccination, other regions that had an absent or modest signal after primary vaccination showed a strong signal after the booster dose (eg, 693-698 residues; **Supplementary Figures 1**, **2A, B**), while region 607 to 612 conversely showed a loss of signal in most samples.

Similar to the PIWAS/PIE findings, a clear reduction in antibody signals toward S-specific motifs by IMUNE was observed at >6 months after primary vaccination (booster day 1) compared with day 57. However, booster vaccination enhanced antibody signals toward S-directed motifs across participant samples (booster day 29; **Figure 2**). While there was some variation in the epitope signals, a strong restoration of most responses was observed after booster vaccination.

### 3.4 Antibody response toward S-specific epitopes after booster vaccination varied by booster vaccine formulation

**Figure 3:**
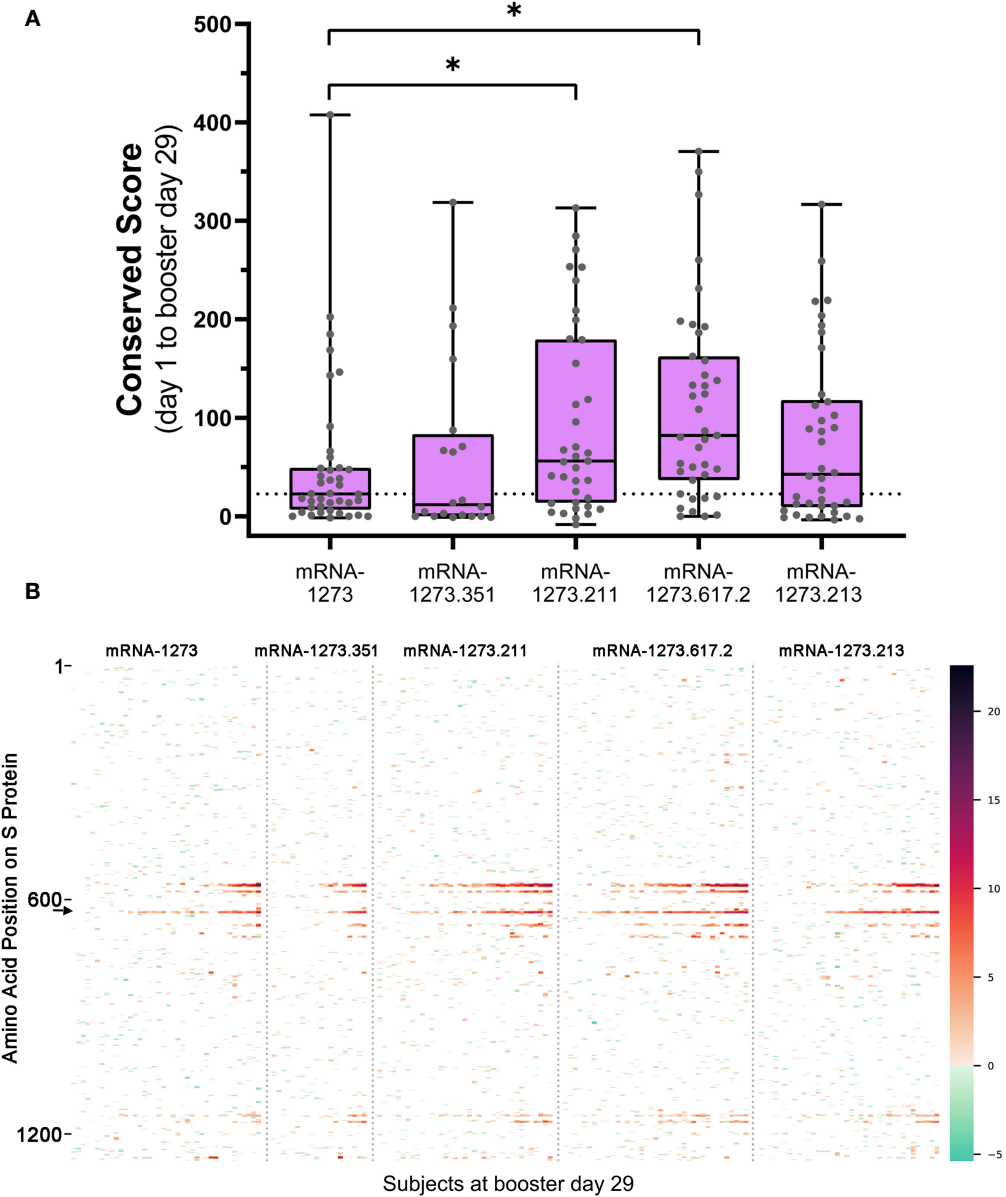
**(A)** Overall antibody signal to SARS-CoV-2 S protein after booster vaccination and **(B)** heatmap visualization of baseline subtracted enrichment signal along amino acid residues of S protein (y-axis) for the 174 participants (columns). **(A)** Baseline subtracted outlier signal at booster day 29 for all post-booster samples was integrated in shared regions identified with PIE and plotted for each sample. The median value for the mRNA-1273 vaccine group was plotted as a dashed line for comparison to the variant-updated booster vaccine groups. A two-sided Mann-Whitney U test was performed for each group compared to mRNA-1273 and yielded P values: 0.485 (mRNA-1273.351), 0.0221* (mRNA-1273.211), 0.000679* (mRNA-1273.617.2), and 0.292 (mRNA-1273.213). Significant P values at confidence level 0.05 are marked with an asterisk. **(B)** Baseline subtracted outlier signal at each amino acid position of S protein for all 174 participants at booster day 29 were plotted by vaccine group. Regions are colored using a gradient based on the magnitude of outlier signal as depicted in the color range bar on the right. Samples were arranged from left to right in order of increasing overall baseline subtracted signal for each booster vaccine. Note that signal strength may vary according to booster formulation. For example, the signal at epitope ~625 (arrow on **B**) was strongest after mRNA-1273.211, mRNA-1273.617.2, and mRNA-1273.213 booster vaccination relative to mRNA-1273. PIE, protein-wide identification of epitopes; S, spike.

We compared the integrated outlier signal identified by PIE for different booster vaccine formulations at booster day 29 and observed significantly elevated antibody signal toward epitopes in samples boosted with mRNA-1273.211 or mRNA-1273.617.2 compared with mRNA-1273 (**Figure 3A**). The differential signals were mostly attributable to five epitopes: 558-KFLPFQQFGR, 577-RDPQTLE, 630-TPTWRVY, 663-DIPIGAG, and 693-IAYTMS (**Figure 3B**). Other notable differences in the signal of shared epitopes on the S protein were also observed across booster vaccine formulations (**Supplementary Figure 1**). For example, the signal at epitope ~625 was strongest after mRNA-1273.211, mRNA-1273.617.2, and mRNA-1273.213 booster vaccination (see an arrow on **Figure 3B**), while the signal toward epitopes ~665 and ~1165 were weaker after an mRNA-1273 booster compared with any variant-updated booster vaccination. Comparatively, a signal toward epitope ~1260 was observed only after booster vaccination with mRNA-1273, mRNA-1273.351, and mRNA-1273.211. These four epitopes on the S protein are invariant across different SARS-CoV-2 strains, including Wuhan-Hu-1 and alpha B.1.1.7, beta B.1.351, delta B.1.617.2, and omicron B.1.1.529 variants. These data suggest that booster vaccination produced differing strengths of antibody responses, dependent on the exact formulation.

### 3.5 Booster vaccination induces antibody signals toward new SARS-CoV-2 S-specific motifs not detected after primary vaccination

**Figure 4:**
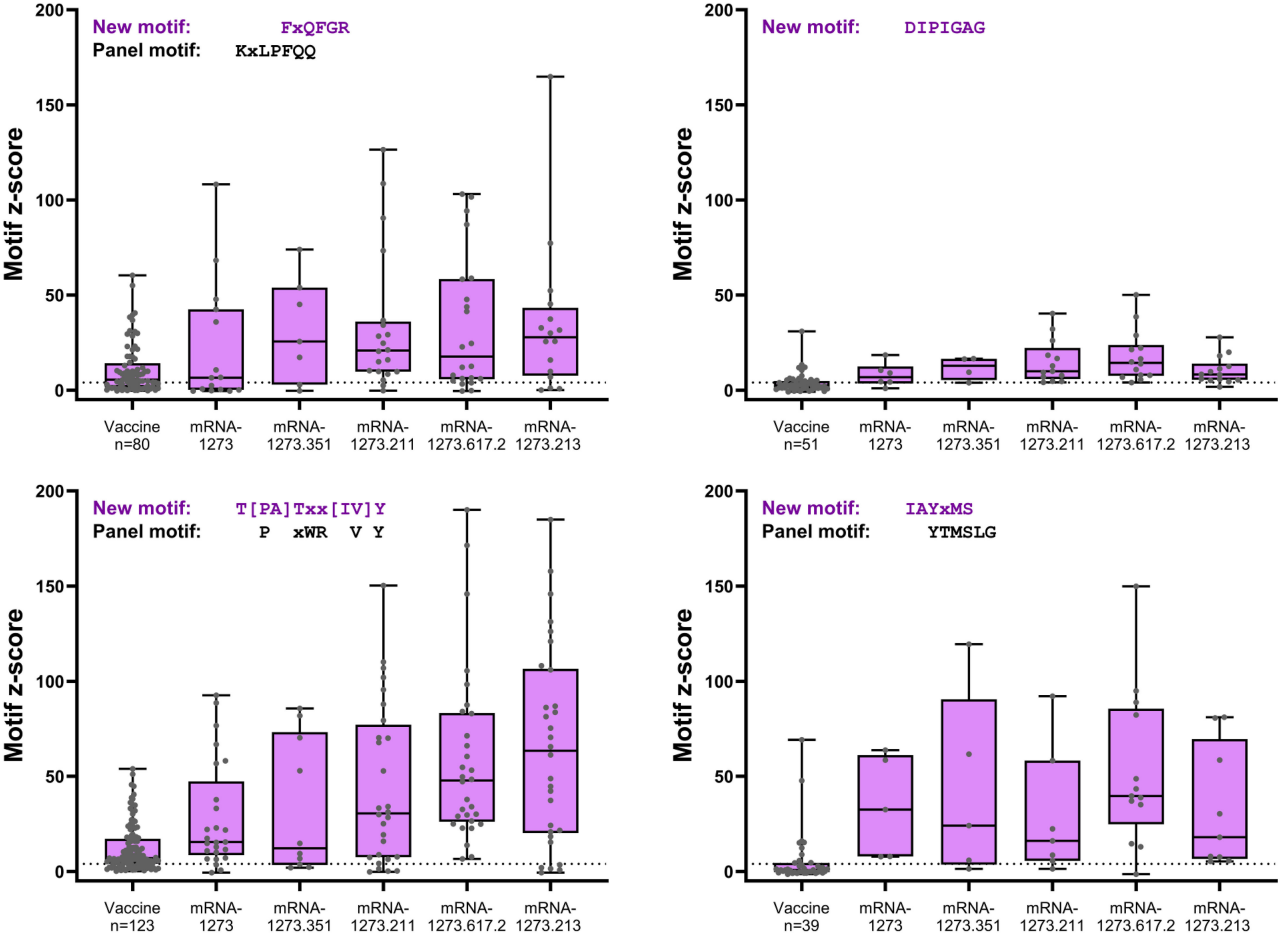
S-specific mapping motifs with increased signal after booster vaccination versus primary vaccination. All matched participant samples with positive motif z-scores (≥4) were plotted for each of the four new motifs with increased signal after booster vaccination (booster day 29) versus after primary vaccination (vaccine). Samples plotted showed positive post-primary vaccination and/or positive post-booster vaccination reactivity. Samples that did not show positive reactivity at either time point are not included. S, spike.

Four S-specific motifs were discovered after booster vaccination that exhibited a combined stronger signal (z-score and percent samples positive) relative to primary vaccination (**Figure 4**; **Table 2**), which was also consistent with findings based on PIWAS/PIE analysis. Two new motif variants, T[PA]Txx[IV]Y and IAYxMS, demonstrated a stronger antibody signal relative to predominant panel motifs after initial vaccination (TPxWRVY and YTxSLG) and a stronger signal after booster versus primary vaccination (21). Of note, the induction of these two motif variants predominantly after booster vaccination is suggestive of possible affinity maturation toward the amino acid content captured in these motifs. Overall, the four new motif variants accounted for a high degree of antibody signal toward the S protein after booster vaccination; if these motifs were omitted from the post-booster vaccination samples, the overall antibody signal (summed z-scores for individual motifs) toward the S protein was then reduced in these samples relative to post-primary vaccination samples. However, inclusion of the four motifs improved the overall signal toward the S protein for both the mRNA-1273.211 and mRNA-1273.617.2 booster vaccines compared with primary mRNA-1273 vaccination. In conjunction with these findings, data suggest that the stronger antibody response after mRNA-1273.211 and mRNA-127.617.2 booster vaccination compared with mRNA-1273 is predominantly due to the presence of these four motifs. An additional motif ([LP]xHHxIH) that did not map to the sequence of the S protein was detected in 18% to 48% of samples after booster vaccination of different formulations, but only 3% of samples had positive scores for this motif (z-scores ≥4) after primary vaccination. These data suggest that booster vaccination elicits a stronger signal for new S-directed motifs post-booster compared to post-primary vaccination.

**Table 2:** Percentage of samples with positive z-scores for four motifs after primary vaccination (day 57) and after booster vaccination (day 29) by booster vaccine formulation.

Primary vaccination (n = 174)	[FML]xQFGR AA 564	T[PA]Txx[IV]Y AA 630	DIPIGAG AA 663	IAYxMS AA 693
	26%	49%	10%	5%
Booster vaccination				
mRNA-1273 (n = 40)	20%	58%	13%	13%
mRNA-1273.351 (n = 20)	25%	40%	15%	20%
mRNA-1273.211 (n = 37)	51%	68%	35%	16%
mRNA-1273.617.2 (n = 39)	49%	77%	36%	33%
mRNA-1273.213 (n = 38)	29%	63%	29%	24%

AA, amino acid.

### 3.6 Residue composition of specific epitopes change after vaccination and identification of shared or individual epitopes in participants

To further examine for shifts in antibody responses to motif epitopes after vaccination, we used the random library within SERA to investigate epitope changes at the individual participant level. As an example of this approach, the antibody signal at epitope region 558 to 569 of one participant in the mRNA-1273.211 booster group had an unexpected steadily increasing signal at this region after primary vaccination, suggesting a possible affinity maturation at this shared epitope (**Supplementary Figure 3A**). In this participant, a proportional increase of the QFGR sequence from day 57 to booster day 29 was observed (**Supplementary Figure 3B**), while the N-terminal signal in this region (558-562) appeared to relatively decrease after primary vaccination. After booster vaccination, the signal at this epitope region appeared qualitatively similar to booster day 1 (>6 months after primary vaccination); however, the signal was quantitatively more robust after the booster dose.

As demonstrated in **Supplementary Figure 4**, most of the linear signals were detected in regions with shared outlier signals, but a measurable proportion of the signals occurred in individual (non-shared) regions for all participants, indicating that individuals possess both shared and unique epitopes toward the SARS-CoV-2 S protein before and after vaccination.

## 4 Discussion

High-resolution characterization of antibody responses to vaccination can provide important insights into the robustness and breadth of humoral immune responses elicited by vaccines. In this study, we profiled antibody epitopes induced by primary mRNA-1273 vaccination and either mRNA-1273 or variant-updated booster vaccination to longitudinally investigate the durability and magnitude of immune responses. Overall, the 2-dose primary series of mRNA-1273 elicited antibody signals toward multiple S protein epitopes, including shared and/or individual (non-shared) epitopes. Booster vaccination with mRNA-1273 or variant-updated vaccines at >6 months after primary vaccination restored waning antibody signals, with four new motifs on the S protein detected, with a stronger signal after boosting than with primary vaccination. Further, the strength of antibody responses after booster vaccination varied by the exact vaccine formulation, with the variant-updated mRNA-1273.211 and mRNA-1273.617.2 vaccines inducing significantly higher antibody signals to target epitopes than the prototype mRNA-1273 vaccine. While many neutralizing antibodies act through binding of RBD, thereby preventing binding of the virus to human ACE2 receptors for viral entry, notably several of the epitopes identified in this study have been found to be neutralizing in a previous study, with different proposed neutralizing mechanisms (33). Taken together, this study has identified key SARS-CoV-2 epitopes targeted by antibodies induced by primary mRNA-1273 and/or multiple booster vaccines.

Based on PIWAS/PIE and motif analyses, multiple distinct epitopes within the SARS-CoV-2 S protein were targeted after mRNA-1273 primary vaccination (day 57) and after booster vaccination (booster day 29) with either mRNA-1273 or a variant-updated vaccine. However, the identity of the targeted epitopes notably changed after booster vaccination, with additional epitope sequences strengthened after the booster dose that were not observed following the primary series. Thus, the targeting of antibodies to additional S-specific epitopes after booster vaccination (either with mRNA-1273 or a variant-updated vaccine) indicate that a broader response is elicited. Further supporting this observation is that the variant-updated mRNA-1273.211 and mRNA-1273.617.2 vaccines induced stronger and broader antibody signals than the original monovalent mRNA-1273 vaccine, which was primarily due to increased booster-specific epitope responses by these two vaccines. Accordingly, although variant-updated vaccines containing omicron subvariants were not tested in this study, we would thus expect authorized omicron-containing vaccines to elicit similar patterns and robust responses to SARS-CoV-2 S-specific epitopes that increase the breadth of antibody response.

Overall, our findings were consistent with the results from immunogenicity studies demonstrating that a primary 2-dose vaccination series of mRNA-1273 induces robust antibody responses against SARS-CoV-2 (34, 35) that wane yet remain detectable over time (8, 9).

Furthermore, we observed strong agreement between PIE and motif discovery analyses on the sequence and regional alignment of targeted epitopes within the SARS-CoV-2 S protein. Specific epitopes observed in this study are generally a subset of those that have been previously characterized in individuals with SARS-CoV-2 natural infection using SERA (21) or other platforms (28, 33, 34). While the functional significance of the observed shared epitopes remains to be fully elucidated, several of these locations have known functional importance and might thus suggest that antibody recognition of these sites could have functional significance. Epitopes 685-RSVAS, 814-KRSFIEDLLF, and 1165-DLGDISGI overlap with the S1-S2 junction cleavage site (682-689), the S2′ cleavage site adjacent to the fusion peptide (FP 814-838), and the heptad repeat region (HR2 1162-1202) involved in fusion, respectively. Notably, the 814-KRSFIEDLLF and 1151-ELDKYF epitopes overlap with “coldspots” that are devoid of amino acid changes in SARS-CoV-2 variants of concern (28). We do note that 558-KFLPFQQFGRD and 577-RDPQTLE epitopes in subdomain 1 have also been reported as neutralizing epitopes that may stabilize the S trimer structure (28, 33, 34). One limitation of this study is that the analysis of the identified epitope signals and magnitude or breadth of the neutralizing antibody response was not performed. This was due to the small sample size and the presence of similarly robust and cross-reactive responses at the time points assessed.

In conclusion, a multifaceted and high-resolution approach was utilized to characterize antibody epitope profiles induced by mRNA-1273 primary as well as monovalent and bivalent booster vaccination. Consistent with immunogenicity studies, mRNA-1273 vaccination robustly activated multiple epitopes within the SARS-CoV-2 S protein that waned over time, but booster vaccination revived signals to the majority of these epitopes while also activating new motifs in the S protein. The strength of antibody signals after booster vaccination was also dependent on the exact booster vaccine formulation, with two variant-updated vaccines having increased signals over the prototype mRNA-1273 vaccine due to increased activity at the newly activated motifs.

## Data availability statement

The original contributions presented in the study are included in the article/**Supplementary Material**. The relevant pseudo code for IMUNE, PIWAS, and PIE are available upon request. Further inquiries can be directed to the corresponding author.

## Ethics statement

The studies involving humans were approved by All participants provided informed consent and all study materials were approved by a central institutional review board (Advarra; Columbia, Maryland); the studies were conducted in accordance with the International Council for Technical Requirements for Registration of Pharmaceuticals for Human Use, Good Clinical Practice Guidelines, and applicable government regulations. The studies were conducted in accordance with the local legislation and institutional requirements. The participants provided their written informed consent to participate in this study. Written informed consent was obtained from the individual(s), and minor(s)’ legal guardian/next of kin, for the publication of any potentially identifiable images or data included in this article.

## Author contributions

BG: Writing – original draft, Writing – review & editing, Formal analysis. EB-J: Writing – original draft, Writing – review & editing, Data curation. RB: Writing – original draft, Writing – review & editing, Data curation. TC: Writing – original draft, Writing – review & editing, Formal analysis. JC: Writing – original draft, Data curation, Writing – review & editing, Conceptualization. KD: Writing – original draft, Data curation, Writing – review & editing. AD: Writing – original draft, Formal analysis, Writing – review & editing. MJ: Writing – original draft, Writing – review & editing, Formal analysis. BM: Writing – original draft, Formal analysis, Writing – review & editing. JR: Writing – original draft, Formal analysis, Writing – review & editing, Data curation. JS: Writing – original draft, Formal analysis, Writing – review & editing. MZ: Writing – original draft, Writing – review & editing, Formal analysis. RW: Writing – original draft, Writing – review & editing, Data curation. SC: Conceptualization, Formal analysis, Writing – review & editing, Writing – original draft. DE: Writing – original draft, Conceptualization, Writing – review & editing. MM: Writing – original draft, Conceptualization, Writing – review & editing, Formal analysis. RPar: Writing – original draft, Conceptualization, Writing – review & editing, Formal analysis. RPaj: Writing – original draft, Conceptualization, Writing – review & editing, Formal analysis.
