# Supplementary material for: Profiling antibody epitopes induced by mRNA-1273 vaccination and boosters
Source: Front Immunol. 2024 Mar 18;15:1285278. doi: 10.3389/fimmu.2024.1285278 (PMC10983613; doi:10.3389/fimmu.2024.1285278)
Supplement: Supplementary file 1 [file DataSheet_1.pdf]

Supplementary Materials

1 Supplementary Methods

1.1 Sample QC

All samples were subjected to multiple analysis metrics for quality control (QC) after sequencing was performed to ensure adequate antibody capture, lack of cross-contamination, and plate-to-plate consistency. All plates used in this study had consistent QC metrics and all samples screened passed internal and well-specific QC metrics. Antibody fingerprinting was used to compare antibody signals across all same-subject samples for determining possible sample mismatch.

2. Supplementary Figures

Supplementary Figure 1 Antibody signals toward SARS-CoV-2 S-specific motifs at each time point after vaccination by booster vaccine formulation. Motif z-scores for individual S protein motifs are shown from before vaccination (day 1) to after booster vaccination (booster day 29) time points. At time points prior to booster vaccination, all samples were plotted as a single point since samples were either baseline (before vaccination) or primary mRNA-1273 vaccination only (error bars represent range of motif median z-scores from participants in each booster vaccine group). After booster vaccination, the plot branches into each of the booster vaccine formulation types to compare motif z-scores in each group. Newly discovered motifs are included for comparison to motif variants already present in the panel. SARS-CoV-2, severe acute respiratory syndrome coronavirus 2.

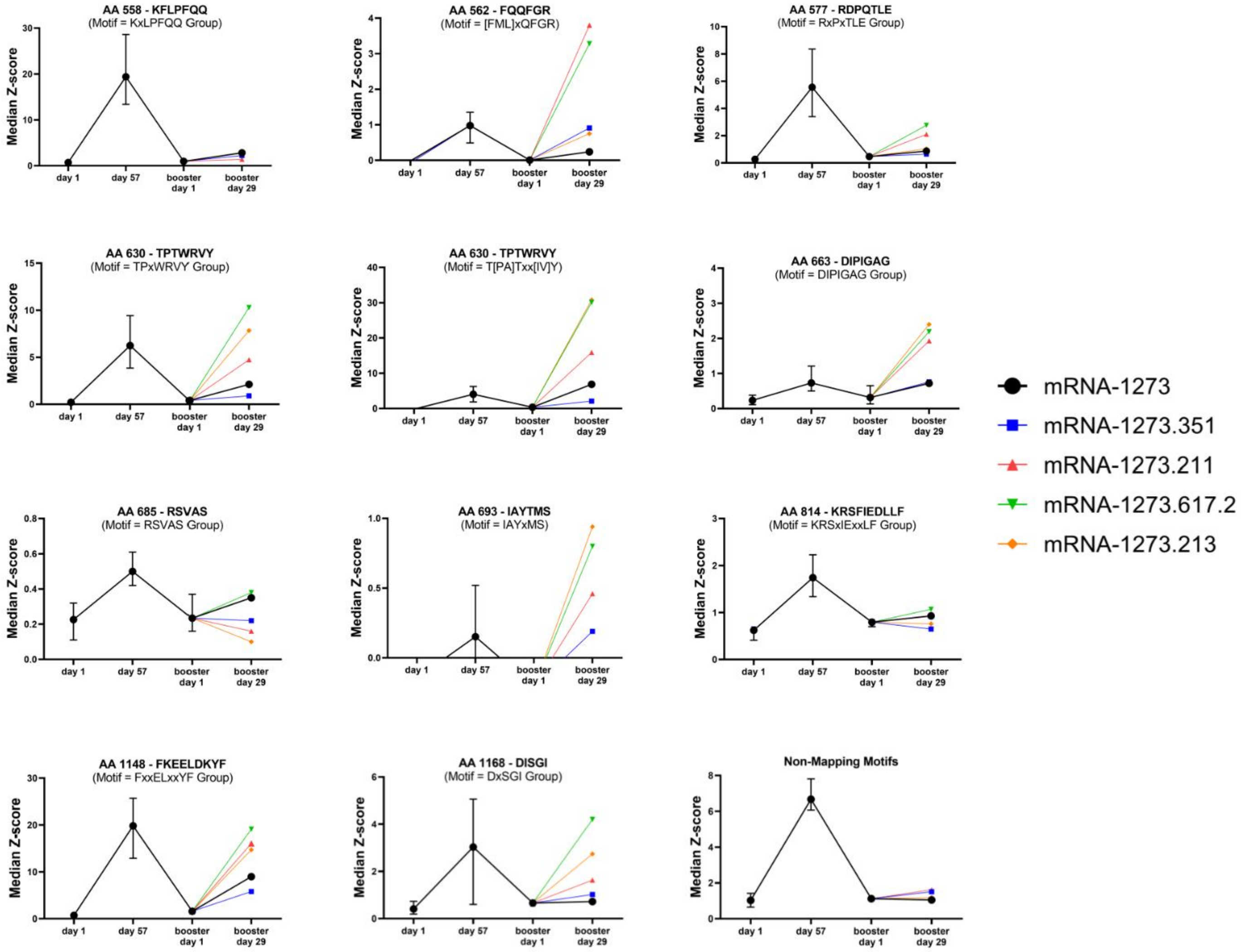

Supplementary Figure 2 Trends in SARS-CoV-2 epitope signals after vaccination. **(A)** Plots demonstrating the 95th percentile PIWAS value for epitope regions 577-583 (booster vaccination restores previously strong epitope signal); 693-698 (booster vaccination enhances a previously weak or absent signal); or 607-612 (strong signal after primary vaccination is not enhanced by booster vaccination) across the study time points. **(B)** Tiling plots of individual samples of the corresponding epitopes (a) at day 57 and booster day 29, where the PIWAS value of a sample is shown along the amino acid position of the SARS-CoV-2 S protein. Note locations 683-698 contain two epitope signal peaks, where the first epitope is at 685-689 and the second epitope is at 693-698. PIWAS, protein-based immunome wide association studies.

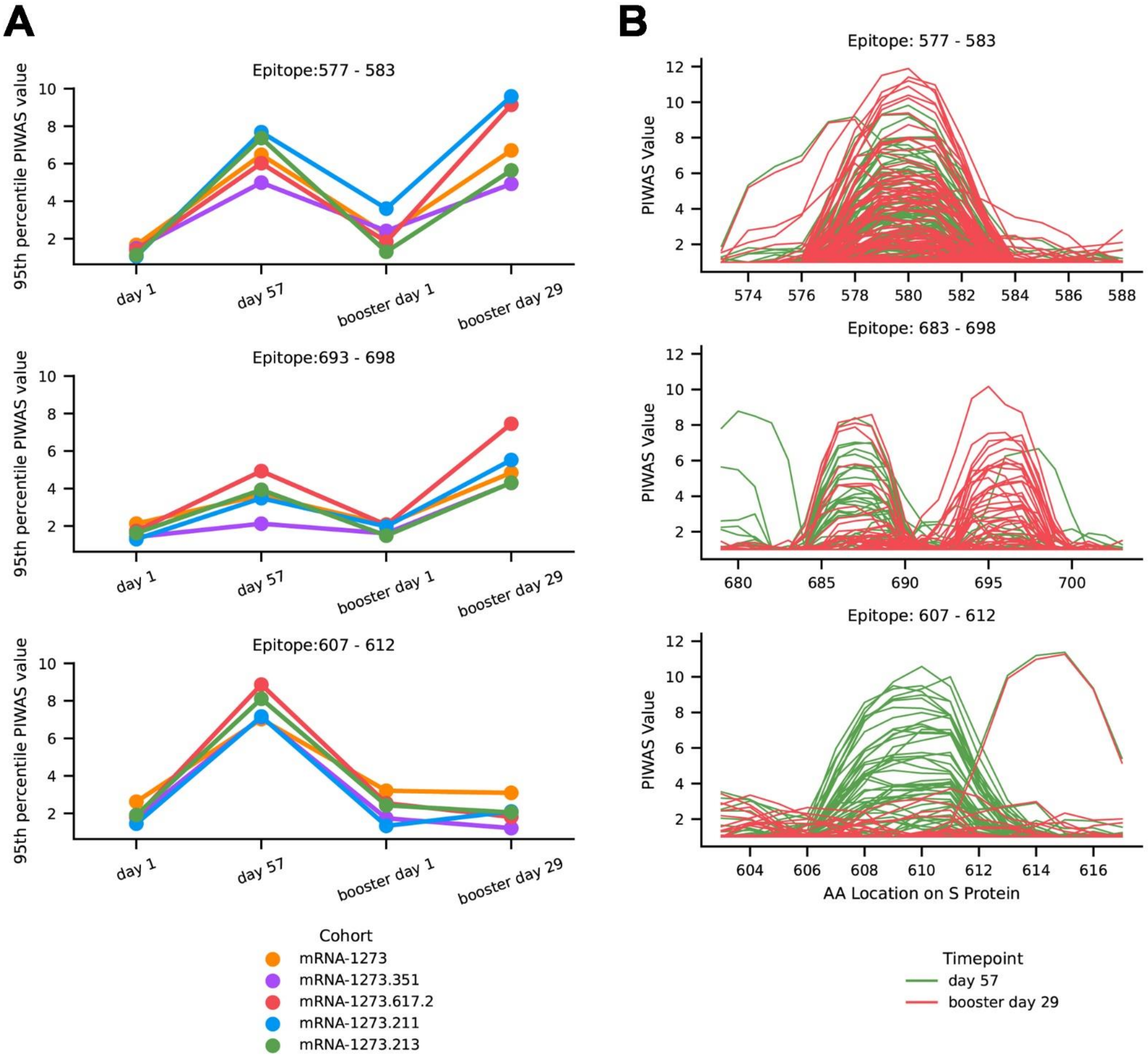

Supplementary Figure 3 High-resolution longitudinal signal from an individual participant. (**a**) PIWAS signal showing longitudinal changes in epitope signal at region 558-569 observed for an individual participant in the mRNA-1273.211 booster group. (**b**) Enrichment logo plots for the same individual participant at region 558-569 from day 1 (before vaccination) to after booster vaccination (booster day 29). Note that baseline is shown but no signal was observed, while booster day 29 was rendered with a larger y-axis for resolution. The non-dominant AA at various positions correspond to additional amino acids that were observed at the respective positions but whose contributions were minor. PIWAS, protein-based immunome wide association studies.

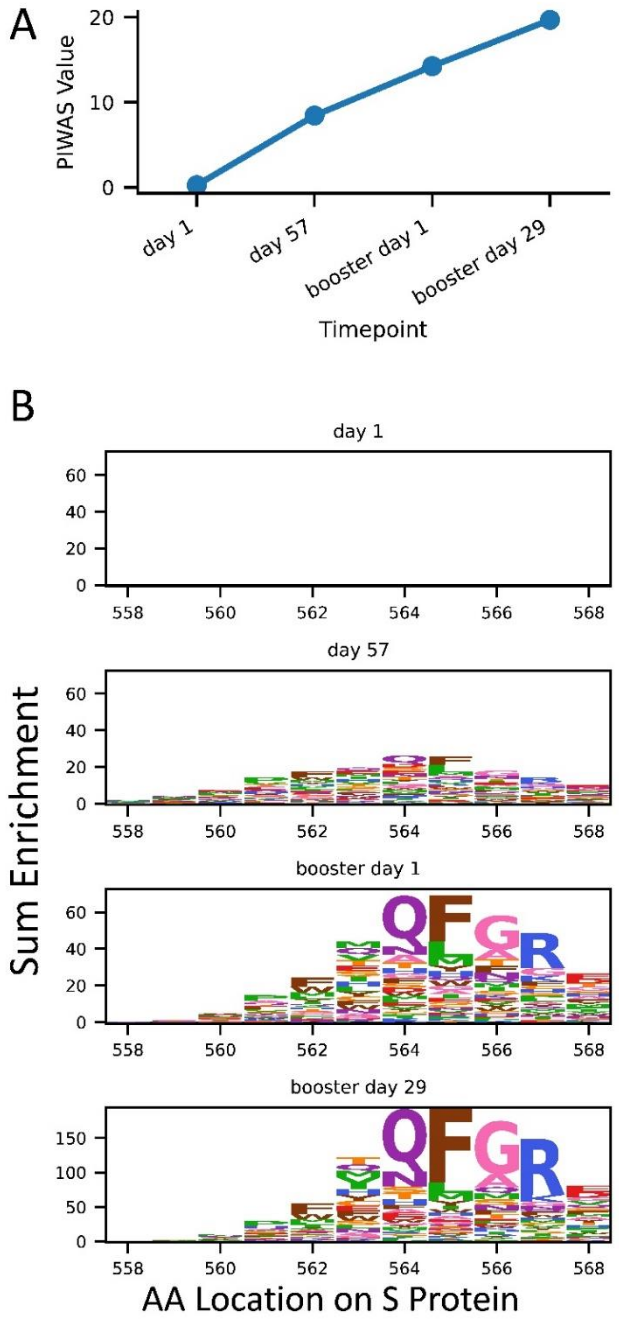

Supplementary Figure 4 Fraction of antibody signal toward shared epitopes after primary vaccination (day 57). Shared scores were calculated for each participant by integrating paired PIE signal at day 57 (with baseline signal removed) in shared amino acid regions identified with PIE. Individual epitope scores were calculated by integrating signal in regions of probable individual epitopes that were defined in all amino acid positions where paired PIE outlier signal increased from baseline to day 57. The ratio of shared score to the sum of shared and individual scores at day 57 is provided for each participant. PIE, protein-wide identification of epitopes.

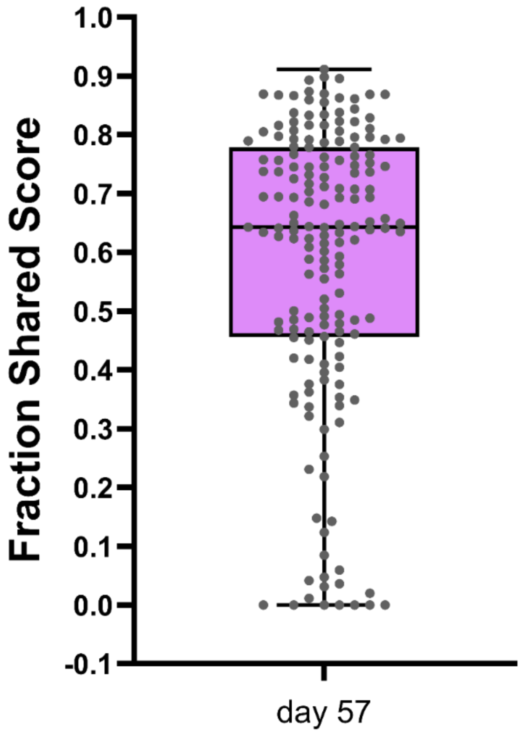

3. Supplementary Tables

Supplementary Table 1 Participant samples.

Booster vaccine	Clinical trial participants	Trial participants, n (samples)
mRNA-1273 (Wuhan-Hu-1)	Enrolled in the open-label interventional phase (Part B) of a phase 2 study (NCT04405076) (14), where participants received a booster dose of mRNA-1273 6 to 8 months after completing the mRNA-1273 primary series	40 (160)
mRNA-1273.351 (beta variant)	Enrolled in the proof-of-concept rollover study (Part C) of a phase 2 study (NCT04405076), where participants who had received two doses of mRNA-1273 in the phase 3 clinical trial (NCT04470427) at least 6 months earlier were rolled over into Part C to receive a booster dose of mRNA-1273.351	20 (80)
mRNA-1273.211 (Wuhan-Hu-1 and beta strains)	Enrolled in Part A of a phase 2 study (NCT04927065), where participants who had received two doses of mRNA-1273 in the phase 3 clinical trial (NCT04470427) at least 6 months earlier were administered a booster dose of mRNA-1273.211	37 (148)
mRNA-1273.617.2 (delta variant)	Enrolled in Part C of a phase 2 study (NCT04927065), where participants who had received two doses of mRNA-1273 in the phase 3 clinical trial (NCT04470427) at least 6 months earlier were administered a booster dose of mRNA-1273.617.2	39 (156)
mRNA-1273.213 (beta and delta strains)	Enrolled in Part D of a phase 2 study (NCT04927065), where participants who had received two doses of mRNA-1273 in the phase 3 clinical trial (NCT04470427) at least 6 months earlier were administered a booster dose of mRNA-1273.213	38 (152)
